# Differences in the Hemolytic Behavior of Two Isomers in *Ophiopogon japonicus In Vitro* and *In Vivo* and Their Risk Warnings

**DOI:** 10.1155/2020/8870656

**Published:** 2020-12-13

**Authors:** Huan-Hua Xu, Zhen-Hong Jiang, Yu-Ting Sun, Li-Zhen Qiu, Long-Long Xu, Xiang-Lin Tang, Zeng-Chun Ma, Yue Gao

**Affiliations:** ^1^Tianjin University of Traditional Chinese Medicine, Tianjin 300193, China; ^2^Department of Pharmaceutical Sciences, Beijing Institute of Radiation Medicine, Beijing 100850, China; ^3^Jiangxi Province Key Laboratory of Molecular Medicine, Nanchang 330006, China; ^4^College of Life Science and Bioengineering, Beijing University of Technology, Beijing 100124, China

## Abstract

Ophiopogonin D (OPD) and Ophiopogonin D′ (OPD′) are two bioactive ingredients in *Ophiopogon japonicus*. Previously published studies have often focused on the therapeutic effects related to OPD's antioxidant capacity but underestimated the cytotoxicity-related side effects of OPD′, which may result in unpredictable risks. In this study, we reported another side effect of OPD′, hemolysis, and what was unexpected was that this side effect also appeared with OPD. Although hemolysis effects for saponins are familiar to researchers, the hemolytic behavior of OPD or OPD′ and the interactions between these two isomers are unique. Therefore, we investigated the effects of OPD and OPD′ alone or in combination on the hemolytic behavior *in vitro* and *in vivo* and adopted chemical compatibility and proteomics methods to explain the potential mechanism. Meanwhile, to explain the drug-drug interactions (DDIs), molecular modeling was applied to explore the possible common targets. In this study, we reported that OPD′ caused hemolysis both *in vitro* and *in vivo*, while OPD only caused hemolysis *in vivo*. We clarified the differences and DDIs in the hemolytic behavior of the two isomers. An analysis of the underlying mechanism governing this phenomenon showed that hemolysis caused by OPD or OPD′ was related to the destruction of the redox balance of erythrocytes. *In vivo*, in addition to the redox imbalance, the proteomics data demonstrated that lipid metabolic disorders and mitochondrial energy metabolism are extensively involved by hemolysis. We provided a comprehensive description of the hemolysis of two isomers in *Ophiopogon japonicus*, and risk warnings related to hemolysis were presented. Our research also provided a positive reference for the development and further research of such bioactive components.

## 1. Introduction

Hemolysis is the rupturing of erythrocytes and the release of their contents into the surrounding fluid. Hemolysis may occur *in vivo* or *in vitro*. Many factors can cause hemolysis, such as intrinsic causes (i.e., defects in the red blood cell (RBC) membrane, defects in hemoglobin [1], defective erythrocyte metabolism [2], Marchiafava-Micheli syndrome [3], etc.) and extrinsic causes (i.e., immune-mediated causes [4], toxicants or chemical reagents, physical factors [5], etc.). Regardless of intrinsic or extrinsic causes, disrupted redox balance is one of the common causes of hemolysis. The RBCs, in addition to their primary role as oxygen carriers, function as redox modulators [6]. However, this modulation ability is limited. Instead, mature erythrocytes, with their absence of protein synthesis and high oxygen-carrying capacity, are particularly susceptible to oxidative damage because they are rich in heme iron and oxygen, which can spontaneously generate H_2_O_2_ and lipid peroxides [7, 8].

**Figure 1:**
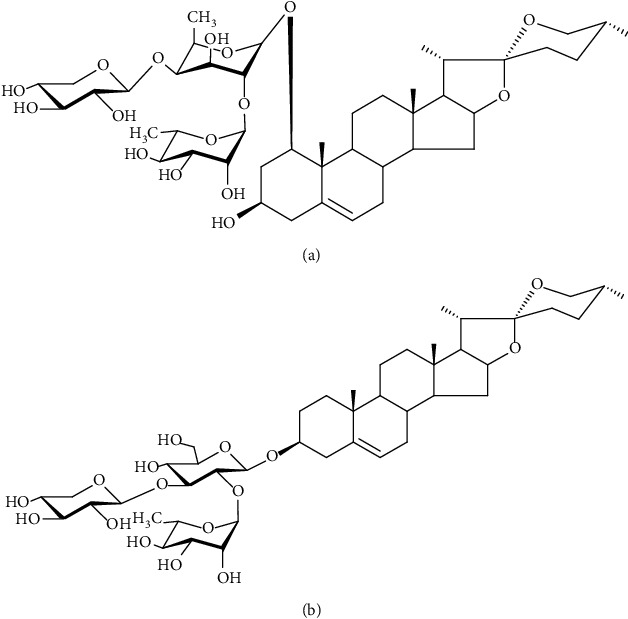
The chemical structure of Ophiopogonin D (OPD) and Ophiopogonin D′ (OPD′). (a) Ophiopogonin D: ruscogenin 3-O-{*α*-L-rhamnopyranosyl (1⟶2)-[*β*-D-xylopyranosyl (1⟶3)]-*β*-D-fucopyranoside}. (b) Ophiopogonin D′: diosgenin 3-O-{*α*-L-rhamnopyranosyl (1⟶2)-[*β*-D-xylopyranosyl (1⟶3)]-*β*-D-glucopyranoside}.Chemical formula: C_44_H_70_O_16_, MW = 854.

*Ophiopogon japonicus* (Thunb.) is a well-known traditional Chinese medicine used to treat cardiovascular and chronic inflammatory diseases [9, 10]. Although it is not included in the medicinal and food dual-use list announced by the National Health Commission of the People's Republic of China, it is allowed to be used in functional foods [11], and in southern provinces of China, *Ophiopogon japonicus* is often used in dietary soups [12]. OPD and OPD′ are two bioactive ingredients in *Ophiopogon japonicus*, and they are isomers of each other (see Figure 1). Previously, published literature on the active monomers in *Ophiopogon japonicus* mostly focused on OPD and its antioxidant effect [13–16]. Since 2017, the literature related to OPD′ has gradually increased and is almost all related to its cytotoxicity [17]. Our mentor's team reported for the first time that OPD′ at a concentration greater than 5 *μ*mol/L can promote H9C2 cell apoptosis through the endoplasmic reticulum stress pathway [18]. Based on its cytotoxic effect, Zongliang et al. [19, 20] developed it as a potential antiprostate cancer agent. However, with the deepening of our studies, we found that the cytotoxicity or other properties, such as the hemolytic properties that we discovered and reported in this manuscript, of OPD′ limit its druggability. Moreover, in this manuscript, we reported that OPD, as an antioxidant, could also cause hemolysis *in vivo*. Despite extensive studies, a comprehensive analysis of hemolysis and drug-drug interactions (DDIs) between OPD and OPD′ is missing, limiting our ability to assess the risks of medicines, functional foods, and diets containing *Ophiopogon japonicus*. Previous profiling studies lacked hemolysis, which necessarily underestimates its adverse effect and restricts analysis in a hemolysis-biased manner.

In this study, we investigated the effects of OPD and OPD′ alone or in combination on the hemolytic behavior *in vitro* and *in vivo*. We identified that there is a huge difference in the hemolysis behavior between OPD and OPD′ and clarified the specific manifestations of the behavior. We further adopted chemical compatibility and multiomics (i.e., metabolomics, lipidomics, and proteomics) methods to explain the different hemolytic behaviors *in vitro* and *in vivo*, and we found that both *in vitro* and *in vivo*, redox imbalance and lipid metabolism disorders played an important role in the hemolysis process. We also inferred that the DDIs between OPD and OPD′ are not limited to the complementarity of physical and chemical properties, and a competitive target related to hemolysis may also exist. Therefore, molecular modeling technology was used to find the common target of the two isomers.

## 2. Materials and Methods

### 2.1. Drugs and Reagents

OPD (F05975, purity: 98% by HPLC) and OPD′ (F581298, purity: 98% by HPLC) were purchased from the Shanghai EFE Biotechnology Co., Ltd. (Shanghai, China). Streptavidin PE conjugate (12-4317-87) was purchased from Thermo Fisher. Biotinamidohexanoic acid N-hydroxysuccinimide ester (B2643, purity ≥ 98%, powder) was purchased from Sigma-Aldrich. 1,1-Diphenyl-2-trinitrophenylhydrazine (DPPH) was purchased from the Tokyo Chemical Industry Co., Ltd. (batch number: 217-591-8). Trolox (Lot No.1218C026, purity: not less than 50%) was purchased from the Solarbio Co., Ltd. (Beijing, China). Water was prepared by double distillation. All other reagents were of analytical or HPLC grade. Water, methanol, acetonitrile, and formic acid were purchased from the Sinopharm Chemical Reagent Co., Ltd. (Beijing, China) and CNW Technologies GmbH (Düsseldorf, Germany).

### 2.2. Animals, Animal Management, and Experimental Design

The erythrocyte suspensions used in the *in vitro* hemolysis test were collected from New Zealand white rabbits purchased from the Beijing Jinmuyang Laboratory Animal Breeding Co., Ltd. (production license: SCXK (Jing)-2015-0005). An *in vivo* hemolysis study was conducted in apparently healthy adult KM mice (18-20 g, male). Animals were purchased from the Beijing Keyu Animal Breeding Center (production license: SCXK (Jing)-2018-0010). The animals used in the multiomics research were Wistar rats that were purchased from the Beijing Vital River Laboratory Animal Technology Co. Ltd. (production license: SCXK (Jing)-2016-0011). All animals were housed in an environmentally controlled breeding room (temperature: 22 ± 2°C, humidity: 50 ± 5%, dark/light cycle: 12/12 h). The animals were provided with standard laboratory food and water. The rabbits were raised in a single cage, and 3 mice and rats were raised per cage. During the experiment, the animals had free access to food and water [21, 22]. The experimental protocols were approved by the Animal Ethics Committee of Academy of Military Medical Sciences (approval No. IACUC-DWZX-2020-684) and were performed in accordance with the guidelines of the National Institutes of Health for the Care and Use of Laboratory Animals. Prior to each experiment, all animals were kept under laboratory conditions for a period of 4 days or more for acclimatization. Since there are multiple independent animal experiments, the experimental design is described in detail under their respective experimental items, such as in Sections 2.3, 2.6, and 2.7.

### 2.3. *In Vitro* Hemolysis Test

The *in vitro* hemolysis test referred to the “Technical Guidelines for the Study of Stimulating and Hemolytic Properties of Traditional Chinese Medicines and Natural Medicines” issued by the State Food and Drug Administration in 2005(Guideline number:[Z]-GPT4-1). Four groups were designed to investigating whether OPD and OPD′ exhibit drug-drug interactions in hemolytic behavior. They were the OPD group, OPD′ group, OPD+OPD′ group (premixed before administration), and OPD⟶OPD′ group (based on the isomers' physical and chemical properties, considering whether preadministration has a protective effect, we set the OPD⟶OPD′ group, in which OPD and OPD′ were used in sequence, 5 min apart). The concentrations of OPD and OPD′ in each group were 0, 5, 10, 20, and 40 *μ*g/mL. After each group of samples were prepared according to the guidelines, they were immediately placed in a 37 ± 0.5°C water bath for 3 h, removed, and centrifuged at 3000 rpm for 5 min, and the supernatant was transferred to a 24-well plate. An ultraviolet spectrophotometer was used to measure the absorbance of each group of samples at 570 nm, calculate the hemolysis rate, and draw the hemolysis rate-concentration curve.

One of the difficulties in the study of active monomers of traditional Chinese medicine is poor water solubility, resulting in dose deviation and inaccurate concentration. Both of OPD and OPD′ suffered from this problem, and our pilot studies have shown that 25% methanol in saline as the solvent can not only completely dissolve OPD and OPD′ but is also safe for RBCs within 4 h of *in vitro* hemolysis experiments. Compared with other solvents, there were no dose deviations, and the results were extrapolated to be more credible clinically.

### 2.4. Effects of Different Solvents or Compatible Drugs on the Hemolytic Behavior of OPD′

To investigate the effects of commonly used clinical solvents on OPD′ hemolytic behavior, saline, glucose (5%), fructose (10%), and mannitol (20%) were selected to dilute the OPD′ stock liquid, in which OPD′ was completely dissolved in methanol (25%), to the following series of concentrations 5, 10, 20, 40, and 80 *μ*g/mL. The hemolysis rate of each sample was determined according to the guidelines, and the hemolysis rate-concentration curve was drawn.

Trolox is a reductive water-soluble vitamin E that protects RBCs from hemolysis due to its oxidative properties. Investigating the effect of the combined use of Trolox and OPD′ on the hemolytic behavior of OPD′ was helpful to explore the reasons for the difference in the hemolytic behavior of the two isomers *in vitro*. OPD′ (40 *μ*g/mL) was selected as the positive control; at this concentration, the hemolysis rate of RBCs *in vitro* was almost 100%. The concentration of OPD′ was fixed and mixed with different concentrations of Trolox solution, and then, the relationship between the change in the hemolysis rate and the concentration of Trolox was revealed. In addition, in the *in vitro* hemolysis test, we discovered that the OPD⟶OPD′ group also exhibited drug-drug interactions. Here, we applied the same procedure as with Trolox to further explore the effect of OPD on OPD′-induced hemolysis.

### 2.5. Reasonable Explanation of the Different Hemolytic Behaviors of OPD′ and OPD *In Vitro*

#### 2.5.1. DPPH Method for the Determination of the Antioxidizability of OPD and OPD′

The DPPH method [23] was first proposed in 1958 and can be used to determine the antioxidant capacity of biological samples *in vitro*. In this study, it was used to investigate whether the difference in the hemolytic behavior between the two isomers is related to the oxidizability and reducibility.

2 mL of 5, 10, 20, and 40 *μ*g/mL OPD and OPD′ samples was added to 5 tubes, followed by the addition of 4 mL of 24 mg/L DPPH ethanol (95%) solution under dark conditions and shaking. After avoiding light at room temperature for 30 min, UV-visible spectrophotometry was used to measure the absorbance (*A*_*i*_) at 517 nm. The same amount of DPPH ethanol (95%) without sample extract was used as the control group, and the absorbance (*A*_0_) of the control group was measured by the same method. Equation (1) was used to calculate the inhibition rate, which represents the antioxidant capacity, and the greater the inhibition rate, the stronger the antioxidant capacity.

(1) $$\begin{array}{c} \text{Inhibition rate}=\frac{A_{0}-A_{i}}{A_{0}}\times 100\%. \end{array}$$

#### 2.5.2. Effect of OPD′ Alone or in Combination with OPD on the SOD, MDA, LDH, GSH, and Na^+^-K^+^-ATPase Content

The experiment was divided into 3 groups: normal control group (NC), OPD′ group, and OPD⟶OPD′ group (OPD and OPD′ were used in sequence 5 min apart). To a 10 mL test tube, 2.5 mL of RBC suspension (20%) and then 2.5 mL of test drugs in different groups were added in turn, with the same volume of solvent added for the control in the NC group. After careful mixing, the mixture was placed in a 37°C constant temperature water bath and incubated for 4 h. After incubation, the test tubes were carefully removed and centrifuged at 1500 rpm (4°C) for 10 min. The supernatant was discarded with a disposable pipette, and 0.2 mL of the lower layer of the RBC pellet was taken for counting cells, to which 3 mL of prechilled purified water was added, followed by mixing and shaking to fully lyse the RBCs. Then, it was stored at 4°C until use. The ELISA kit instructions were followed to determine lactate dehydrogenase (LDH) enzyme activity and the contents of malondialdehyde (MDA), superoxide dismutase (SOD), glutathione (GSH), and Na^+^-K^+^-ATPase in the cytoplasm of each group of erythrocytes [24, 25].

### 2.6. *In Vivo* Hemolysis Test

Biotin labeling, as an alternative nonradioactive approach for the determination of RBC survival, is widely accepted [26, 27]. In this study, 30 mg/kg biotinamidohexanoic acid N-hydroxysuccinimide ester solution was injected via the tail vein to label RBCs. After 1 h, to determine the efficiency of biotin labeling, blood samples were collected from each animal, and streptavidin PE conjugate was used to bind biotin, which was used to label RBCs for flow cytometric analysis. Animals with a labeling efficiency greater than or equal to 95% were selected for RBC lifespan analysis.

Fifteen mice were randomly assigned to 3 groups: normal control group (NC), OPD group, and OPD′ group. According to the limit of the clinical hemolysis rate (5%) and the results obtained from the *in vitro* hemolysis test, the plasma concentration of OPD′ should not exceed 5 *μ*g/mL. The doses of OPD and OPD′ in each group were defined as 0.25 mg/kg; at this dose, the configuration of the drug was free of organic reagents. All drugs were administered by intravascular injection for 30 days. Changes in the percentage of labeled RBCs with the administration time can be used to estimate the RBC lifespan. The first blood sample analysis should be completed within 3 days after administration, and the subsequent analysis interval was based on the first analysis results, but did not exceed 7 days.

### 2.7. Proteomics Methods to Explore the Potential Mechanism of OPD- and OPD′-Induced Hemolysis *In Vivo*

Twenty-two rats were randomly assigned to 3 groups: the normal control group (NC, *n* = 6), OPD group (*n* = 8), and OPD′ group (*n* = 8). All drugs were administered by intravascular injection for 14 days. According to the limit of the clinical hemolysis rate (5%) and the results obtained from the *in vitro* hemolysis test, the doses of OPD and OPD′ in each group were defined as 0.25 mg/kg. Plasma was prepared by centrifugation for metabolomics and lipidomics analysis. Both metabolomics and lipidomics analyses were performed on a UPLC-MS/MS system tandem with different mass spectrometers and separated under different LC conditions (unpublished manuscript).

At the same time, the nonanticoagulated whole blood of each group was prepared into RBC suspensions according to the guidelines (Guideline number: [Z]-GPT4-1). Subsequently, pure water was added to fully lyse the RBC membrane, followed by centrifugation. The supernatant was discarded, and the precipitate was washed 2-3 times repeatedly. Then, the precipitate was the prepared blood ghost, i.e., the RBC membrane. Fifty microliters of sample lysate was added, and then, protease inhibitor PMSF was added to make the final concentration 1 mM. The solution was then boiled for 10 min and centrifuged at 12000 g for 10 min at room temperature. The supernatant was centrifuged again, and the supernatant is the total protein solution of the sample, with the protein concentration determined by the BCA method. After protein quantification, 100 *μ*g of each sample was placed into a 10 K ultrafiltration tube, and the filter-aided sample preparation (FASP) method [28] was used to enzymatically digest the protein. For tandem mass tag (TMT) labeling, 41 *μ*L of anhydrous acetonitrile was added to a TMT^6^ [29] reagent vial at room temperature. The reagents were dissolved for 5 min and centrifuged. Then, 41 *μ*L of the TMT^6^ label reagent was added to each 100 *μ*L sample for mixing. The tubes were incubated at room temperature for 1 h. Finally, 8 *μ*L of 5% hydroxylamine was added to each sample and incubated for 15 min to terminate the reaction. The prepared sample was first separated by reverse-phase chromatographic separation, and the sample in the period of 8-60 min was collected. Then, the collected samples were further separated by a HPLC system and detected by mass spectrometry. The data were analyzed by Proteome Discoverer™ software (Version 2.2, Thermo, USA), and the databases utilized were UniProt-proteome_UP000002494-Rattus norvegicus (Rat) (Strain Brown Norway). Detailed proteomic analysis methods can be seen in the supplementary materials (see Method S1).

### 2.8. Molecular Modeling to Find the Common Hemolysis Target of OPD and OPD′

The bioassay of OPD′ and OPD indicates that they will result in hemolysis *in vitro* and *in vivo*. To predict the putative targets and conduct pathway enrichment analysis for OPD′ and OPD, the targets associated with hemolysis were collected from famous databases such as UniProt. Putative target prediction was based on the SEA (similarity ensemble approach) [30] approach. This method is based on the molecular fingerprint to calculate the similarity between the query molecule and the corresponding ligand molecule in the target database. ChEMBL17 was used as the target database. RDKit ECFP4 (1024 bits) was used as the molecular fingerprint. Pharmaceutical target seeker (PTS) [31] was used as a shape-based approach. In PTS, the weighted Gaussian algorithm (WEGA) [32], which is based on molecular three-dimensional conformation superposition, was used to calculate the 3D similarity between the query molecule and the corresponding ligand molecule in the target database. The Reactome Pathway Database (https://reactome.org) was used for pathway analysis for the predicted potential targets of OPD′ and OPD.

### 2.9. Statistical Analysis

SAS version 9.1 (SAS Inc. USA) was used to perform the statistical analyses. All data are expressed as the mean ± SD. For comparisons between two groups, Student's *t*-test was used. For comparisons among three or more groups, the data were analyzed using a one-way analysis of variance (ANOVA). For all analyses, a *p* value of <0.05 was considered to indicate statistical significance and a *p* value of <0.01 was considered to indicate extreme statistical significance. GraphPad Prism (version 6.0) was used to draw the statistical figures.

## 3. Results and Discussion

### 3.1. *In Vitro* Hemolytic Behavior of OPD and OPD′ Is Inconsistent

**Figure 2:**
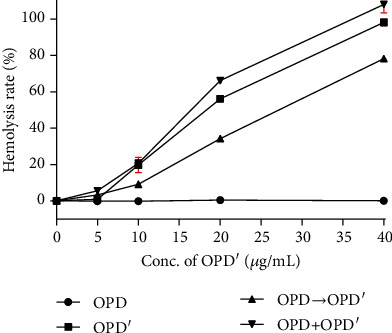
*In vitro* hemolytic behavior of OPD and OPD′. The error bars represent the standard deviation of measurements for 4 parallel samples in four separate groups (*n* = 4).

The hemolytic behaviors of the 4 test groups (i.e., OPD, OPD′, OPD⟶OPD′, and OPD+OPD′) are presented in Figure 2. The curve shows that OPD has no erythrocyte hemolysis in the concentration range of 0-40 *μ*g/mL. In contrast, the RBCs were rapidly hemolyzed within 15 min after the administration of OPD′, and the hemolysis rate was linearly related to the concentration of OPD′. According to the clinical guidelines, the unacceptable hemolysis rate is 5% (Guideline number: [Z]-GPT4-1); therefore, the concentration of OPD′ should not exceed 5 *μ*g/mL. More interesting data came from the other two groups, and the hemolytic behavior of OPD′ was weak when OPD and OPD′ were used in sequence (group OPD⟶OPD′). At a concentration of 20 *μ*g/mL, the hemolysis rate of the OPD′ group was approximately 10% lower than that of the OPD+OPD′ group. However, the OPD⟶OPD′ group's data suggest that OPD plays a role in protecting erythrocytes from being destroyed by OPD′. Obviously, the hemolytic behavior of each group is inconsistent, which provides much evidence for subsequent research.

### 3.2. Different Solvents or Compatible Drugs Affect the Hemolytic Behavior of OPD′*In Vitro*

**Figure 3:**
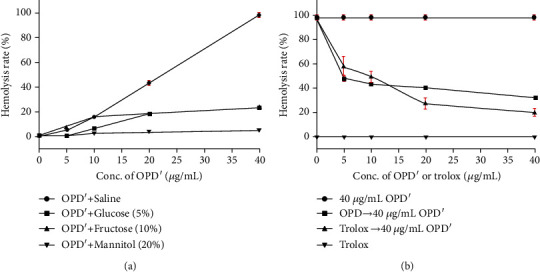
Effects of different solvents or compatible drugs on hemolytic behavior of OPD′. (a) The commonly used clinical solvents. (b) The compatible drugs, OPD, and Trolox. The error bars represent the standard deviation of measurements for 4 parallel samples in four separate groups (*n* = 4).

The research results have shown that different solvents or compatible drugs have a significant effect on OPD′-induced hemolysis. As shown in Figure 3(a), compared with saline, glucose (5%), fructose (10%), and mannitol (20%) alleviated hemolysis caused by OPD′, and mannitol (20%) had the most obvious alleviating effect. For example, when mannitol (20%) was used as the solvent, the hemolysis rate of OPD′ (40 *μ*g/mL) was 4.67% ± 0.42%, and this value was 98.15% ± 1.80% when saline was used as the solvent; therefore, the hemolysis rates was reduced by 93.48%. Correspondingly, the hemolysis rates of glucose (5%) and fructose (10%) were 22.53% ± 1.26% and 23.36% ± 1.07%, respectively.

As shown in Figure 3(b), the compatible drug Trolox did not cause hemolysis in a series of concentrations, and 40 *μ*g/mL OPD′ maintained a hemolysis rate close to 100% (98.15% ± 1.80%). When a series of concentrations of OPD and Trolox were pretreated with erythrocytes, the high hemolysis rate caused by OPD′ (40 *μ*g/mL) was decreased, and the degree of reduction was linearly related to the concentration of OPD and Trolox. In the range of 5-10 *μ*g/mL, the decreasing effect of OPD was better than that of Trolox. However, in the range of 20-40 *μ*g/mL, the decreasing effect of OPD was weakened compared with that of Trolox. The better the decreasing effect, the stronger the ability to protect erythrocytes.

### 3.3. Relationship between Different Hemolytic Behaviors of OPD and OPD′ and the Redox Balance

**Figure 4:**
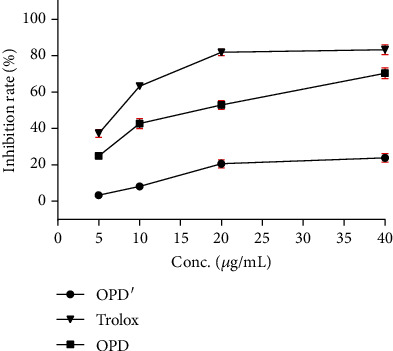
The scavenging ability of OPD, OPD′, and Trolox in different concentrations on DPPH free radicals. The error bars represent the standard deviation of measurements for 4 parallel samples in three separate groups (*n* = 4).

According to the results of Figures 2 and 3, OPD, Trolox, glucose (5%), and fructose (10%) can all alleviate the hemolysis rate caused by OPD′. Among them, Trolox, glucose (5%), and fructose (10%) are all reductive substances. To explore whether the difference in the hemolysis behavior of OPD and OPD′ is related to reductive or oxidative properties, the DPPH method was adopted. As shown in Figure 4, OPD′ has almost no DPPH free radical scavenging ability. In contrast, the DPPH free radical scavenging ability of OPD became stronger as the concentration of OPD gradually increased. However, compared with the positive control drug Trolox, OPD is not as good as Trolox in scavenging DPPH free radicals. Therefore, OPD′ has almost no reducibility, while OPD has a certain degree of reducibility, which could reasonably explain why OPD has the ability to alleviate hemolysis caused by OPD′ similar to Trolox, glucose (5%), and fructose (10%).

**Figure 5:**
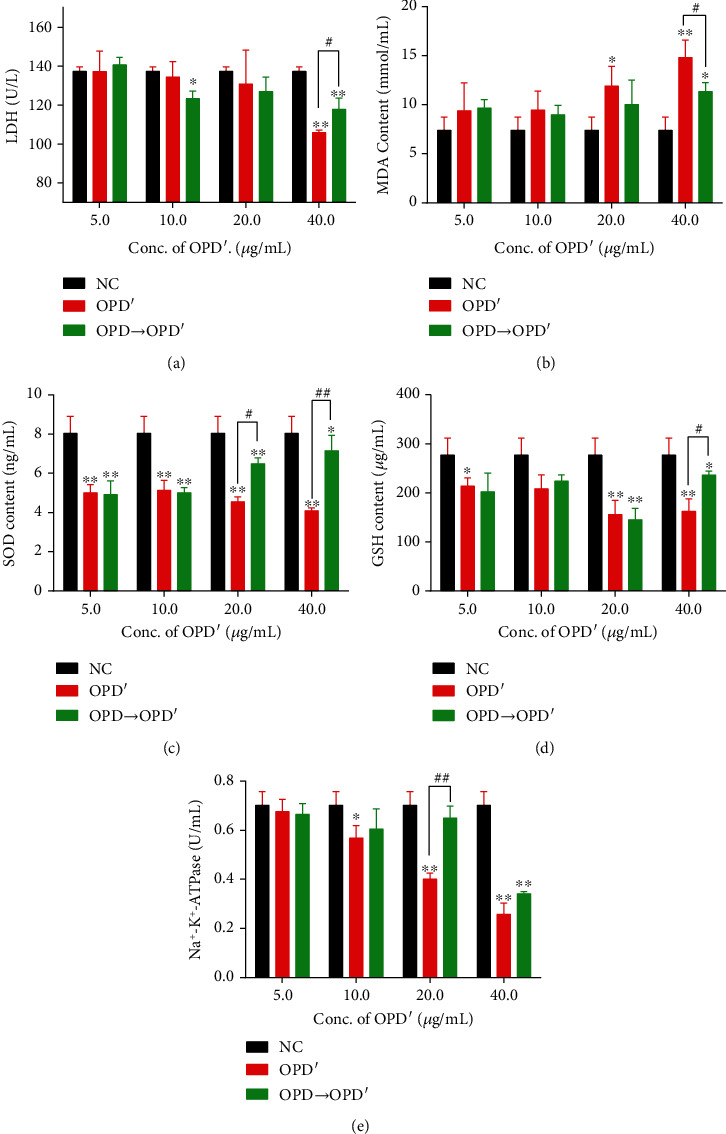
Effects of OPD′ and OPD pretreated (OPD⟶OPD′) on LDH, MDA, SOD, GSH, and Na^+^-K^+^-ATPase content: (a) LDH content, (b) MDA content, (c) SOD content, (d) GSH content, and (e) Na^+^-K^+^-ATPase content. The error bars represent the standard deviation of measurements for 3 parallel samples in three separate groups (*n* = 3). ^∗^Compared with the NC group, the statistical value was significant (*p* < 0.05); ^∗∗^compared with the NC group, the statistical value was very significant (*p* < 0.01); ^#^compared with the OPD′ group, the statistical value was significant (*p* < 0.05); ^##^compared with the OPD′ group, the statistical value was very significant (*p* < 0.01).

As shown in Figure 5(a), the LDH content in the erythrocyte cytoplasm gradually decreased as the OPD′ concentration increased compared with that in the NC group. At concentrations of 10 *μ*g/mL and 20 *μ*g/mL, the difference was significant (*p* < 0.05), and at a concentration of 40 *μ*g/mL, the difference was very significant (*p* < 0.01). When OPD was used to pretreat erythrocytes at a concentration of 40 *μ*g/mL, the LDH content in the cytoplasm of erythrocytes increased significantly (*p* < 0.05) compared with that in the OPD single-use group. After treatment with OPD′, the MDA content in the cytoplasm of RBCs increased, which was positively correlated with the OPD′ concentration. Pretreatment with OPD alleviated the increase in the MDA content caused by OPD′ (Figure 5(b)). Trends similar to the change in the LDH content also appeared in the SOD, GSH, and Na^+^-K^+^-ATPase content (Figures 5(c)–5(e)). After treatment with OPD′, the contents of SOD, GSH, and Na^+^-K^+^-ATPase in the erythrocyte cytoplasm all showed a dose-dependent decrease, and this decrease was reversed or alleviated by pretreatment with OPD to a certain extent.

From the results of the *in vitro* hemolysis test, the effects of solvents and compatible drugs on the hemolysis test, and the detection of redox balance-related factors, it is speculated that the hemolysis caused by OPD′*in vitro* may be related to the destruction of the redox balance. LDH is a marker enzyme that exists in the cytoplasm of cells and can sensitively reflect the degree of cell membrane damage [33]. MDA is a naturally occurring product of lipid peroxidation and prostaglandin biosynthesis that is mutagenic and carcinogenic [34], and the MDA content reflects the degree of lipid peroxidation in erythrocytes. SODs constitute a very important antioxidant defense against oxidative stress in the body [35]. The enzyme acts as a good therapeutic agent against reactive oxygen species-mediated diseases. GSH plays an important role in the body's biochemical defense system. In addition to eliminating free radicals generated by metabolism [36], GSH can also improve the body's immunity. Both SOD and GSH play a very important role in maintaining the redox balance. After treatment with OPD′, the erythrocyte membrane was damaged, resulting in increased membrane permeability and the leakage of cell content such as LDH. The MDA content was significantly increased, and both the SOD and the GSH content were significantly decreased. Na^+^-K^+^-ATPase is an important enzyme for erythrocytes to complete energy metabolism [37]. It actively transports K^+^ and Na^+^, thereby maintaining the balance of osmotic pressure on both sides of the cell membrane. In our study, after treatment with OPD′, its enzyme activity was suppressed.

Therefore, based on the experimental results, we can infer that the hemolytic behavior of OPD′ in RBCs manifests as oxidative damage. By inhibiting the activities of SOD and GSH, it reduces the ability of RBCs to scavenge superoxide anion free radicals, leading to increased membrane permeability, aggravating the degree of lipid peroxidation of RBC membranes, resulting in increased MDA content, and further aggravating the oxidative damage of cell membranes. Increased membrane permeability also induced LDH leakage, which resulted in a decreased LDH content in the cytoplasm of erythrocytes. OPD′ can also inhibit the activity of ATPase on the RBC membrane, affect its material transport and energy metabolism, and lead to changes in the structure and function of the membrane. Eventually, erythrocytes were ruptured, and hemolysis occurred. However, OPD can reverse the hemolysis caused by OPD′ to a certain extent because of its reducibility.

### 3.4. Both OPD and OPD′ Caused Hemolysis *In Vivo*

The differences between the in vitro hemolysis behavior of OPD and OPD′ and the drug-drug interactions (DDIs) based on the hemolysis behavior of the two isomers were very peculiar, which makes us eager to explore the hemolysis behavior and DDIs of these two isomers *in vivo*. We have used a variety of methods to study the hemolytic behavior of OPD and OPD′*in vivo*, such as single-dose- and multiple-dose-based acute toxicity tests (single-dose or 14 days) and subacute toxicity tests (30 days), and the experimental animals include rats and mice [38]. The data in unpublished manuscripts show that multiple doses of OPD and OPD′ both caused hemolysis *in vivo*, which was manifested by a decrease in the HGB content, a decrease in the RBC count, and a significant increase in the absolute value and percentage of RET. Correspondingly, the results related to hemolysis were also observed in the pathological changes of organs and the animal body weight. Urine test results also indicate the occurrence of intravascular hemolysis.

**Figure 6:**
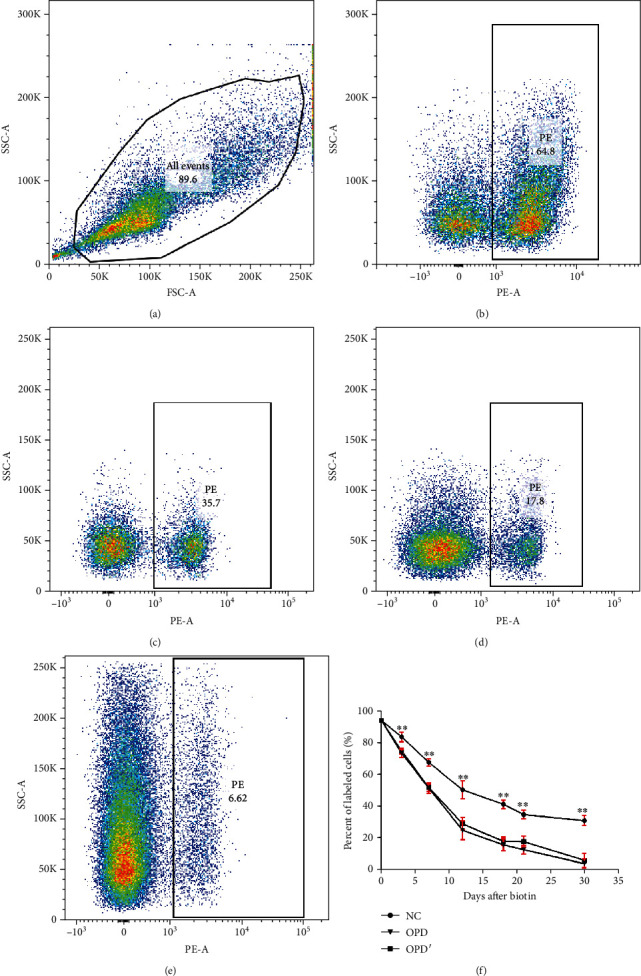
Effects of OPD′ and OPD on erythrocyte life span. (a) and (b) exhibited the flow cytometry analysis workflow; (b) 7^th^ day, NC group; (c) 12^th^ day, OPD group; (d) 21^th^ day, OPD group; (e) 30^th^ day, OPD′ group; (f) relationship between percent of labelled cells and days after biotin. The error bars represent the standard deviation of measurements for 5 parallel samples in three separate groups (*n* = 5). ^∗∗^Compared with the OPD and OPD′ group, the statistical value was very significant (*p* < 0.01).

Although these indicators indicate the occurrence of hemolysis, they are not the gold standard examination for the diagnosis of hemolysis. The essential feature of any hemolytic disorder is the shortened RBC lifespan [39, 40]. As shown in Figure 6(f), administering OPD and OPD′ through tail vein injection for 30 consecutive days significantly shortens the lifespan of RBCs. At the same time point (i.e., 3^rd^, 7^th^, 12^th^, 18^th^, 21^st^, and 30^th^), compared with the NC group, the percentages of labeled cells in both the OPD and OPD′ groups were significantly decreased (*p* < 0.05 or 0.01). These results confirmed that both OPD and OPD′ caused hemolysis *in vivo*. Thus far, we not only discovered the hemolysis of OPD′*in vitro* but also found the hemolysis of OPD *in vivo*.

There are some possible speculations for this phenomenon; one is that OPD is biotransformed into OPD′ or its analogues in animals, and the other one is that both OPD and OPD′ were metabolized into more activated forms for hemolysis, but the speculations lack sufficient data. Based on the speculations, we studied the possibility of the conversion between OPD and OPD′ at both the *in vivo* and *in vitro* levels, and the results did not find any signs of such conversion. We hypothesized that the inconsistency of the hemolytic behavior of OPD *in vivo* and *in vitro* may be related to its metabolites, and related research is ongoing.

### 3.5. Multiomics Studies Reveal the Underlying Mechanism of Hemolysis Caused by OPD and OPD′

Our previous results in an unpublished study demonstrated that changes in endogenous differential metabolites and differential lipids, enrichment of differential metabolic pathways, and correlation analysis all showed that the causes of the hemolysis of OPD and OPD′ are closely related to disorders of phospholipid metabolism.

**Figure 7:**
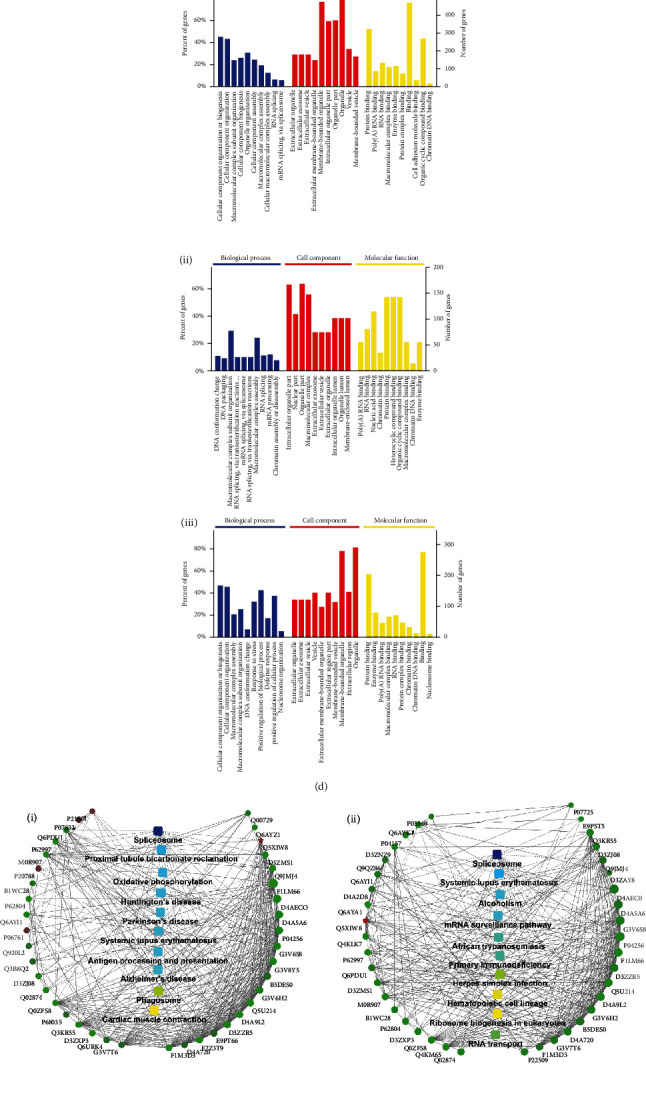
Proteomics results of OPD and OPD′. (a) Venn plot of three compared sets. (b) Up- or downregulated differential proteins in three compared sets. (c) KEGG bubble plot of three compared sets. (d) Top 10 biological processes, cell components, and molecular functions of three compared sets. (e) PPI network plots of three compared sets. (i) NC-OPD-compared group, (ii) NC-OPD′-compared group, and (iii) OPD-OPD′-compared group.

In this study, we focused on the proteomics results of blood ghosts. Based on the screening criteria (i.e., fold‐change > 1.5 or <0.6), 2905 credible proteins were obtained. Compared with the NC group, 636 and 270 proteins were differentially expressed between the OPD and OPD′ groups, respectively, and 364 proteins were differentially expressed between the OPD and OPD′ groups. As shown in Figure 7(a), a Venn plot shows the logical relationships between the three compared sets. In Figure 7(b), the number of up- or downregulated differential proteins is displayed. In Figure 7(c), a bubble plot of KEGG showed that the related enriched significant pathway terms resulted in three compared sets. In Figure 7(d), the top 10 biological processes, cell components, and molecular functions of the three compared sets are displayed. Under OPD intervention, blood ghost proteomics results suggested that biological processes were significantly enriched in the cellular component, subunit or organelle organization, biogenesis, and assembly. However, when treated with OPD′, biological processes were different and enriched in DNA conformation changes and packaging, RNA splicing, mRNA splicing, and processing. This provides a new perspective for understanding the interactions of OPD′ and OPD with RBCs. Cell component analysis indicated that extracellular organelles, exosomes, extracellular membrane-bound organelles, and vesicles were significantly changed in the NC vs. OPD comparison set. In the NC vs. OPD′ comparison set, in addition to extracellular organelles, exosomes, and vesicles, intracellular organelle parts and lumens were significantly changed. At the molecular function level, OPD and OPD′ were similar, and they were both enriched in enzyme, protein, and RNA binding. To realize these analyses and visualize the protein interactions between differential proteins and the KEGG pathway, PPI network plots were selected and are shown in Figure 7(e).

Proteomics analysis provided a comprehensive description of protein changes in OPD- and OPD′-treated erythrocytes, but it did not specify the targets for the next step. Differential protein analysis suggested that hemolysis caused by OPD and OPD′ may contribute to lipid metabolic disorders (e.g., Q5M872 [41] and Q5XIT9), mitochondrial energy metabolism (e.g., D3ZAQ0 [42] and Q03344), high mobility group protein binding (e.g., P63159 and Q4KLJ0 [43]) and cytoskeletal (e.g., Q6IFU7 [44, 45], Q6IFU9, and A0A0G2JST3). These proteins are very valuable research objects.

### 3.6. Some Potential Targets Related to Hemolysis Reveal the Difference in Hemolytic Behavior between OPD and OPD′

**Figure 8:**
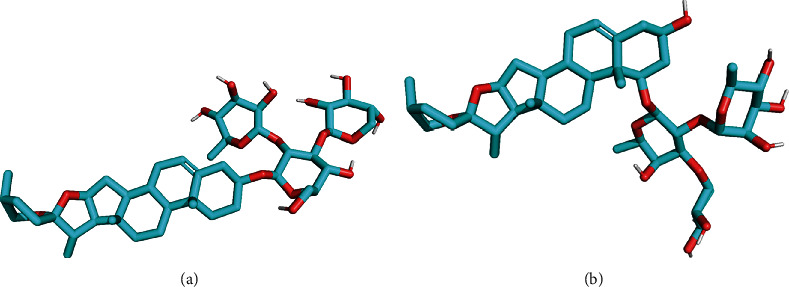
Three-dimensional molecular structures of OPD and OPD′: (a) OPD′; (b) OPD.

We first built the three-dimensional molecular structures of OPD and OPD′ (see Figure 8) and predicted the putative targets based on the SEA approach and shape-based approach, respectively.

The putative targets of OPD and OPD′ predicted by the SEA method are shown in Table 1. Among the 12 targets, the target predicted by the OPD′ structure accounted for 100%, while the target predicted by the OPD structure only accounted for 5/12. These 5 targets are indexed as 2, 6, 8, 9, and 10, respectively.

The top 10 targets predicted by the shape similarity method are shown in Table 2.

The putative targets predicted by the shape similarity method were combined with all the putative targets predicted by SEA. These putative targets were then compared with the collected hemolysis-related targets (see Table S1). The results illustrate that the target “solute carrier organic anion transporter family member 1B3” (Q9NPD5) is the potential target for OPD′. Q9NPD5 was submitted to the Reactome Pathway Database for pathway analysis. Detailed pathway information is shown in Table 3.

Q9NPD5, also known as OATP1B3, is an anion transporter. It has been proven that the hepatic uptake of OPD and OPD′ is mediated by organic anion-transporting polypeptides (OATPs) [46–48]. This report, on the one hand, confirmed the reliability of the molecular modeling results; on the other hand, it pointed out that OPD and OPD′ can affect the transporting activities of OATP1B1 and OATP1B3 in a substrate-dependent manner. Several reports in the literature have shown that the inhibition of organic anion transporting polypeptides OATP1B1 and OATP1B3 might lead to hyperbilirubinemia [49, 50]. This may be one of the reasons why OPD and OPD′ cause hemolysis *in vivo*, and it is also a potential hemolysis prevention target worthy of further study.

In addition to Q9NPD5, several other targets that are not directly related to hemolysis are also worthy of attention, such as AT1A1 (Table 1, Index 2), which affects Na^+^-k^+^-ATPase activity, and its effect on hemolysis has been discussed in the previous paragraph. DHCR7 (Table 1, Index 6), EBP (Table 1, Index 8), and ERG2 (Table 1, Index 9) proteins encoded by these genes were all involved in the cholesterol biosynthetic pathway, which is part of steroid biosynthesis [51–53]. The interaction between OPD or OPD′ and these proteins will further affect the integrity of the cell membrane structure, which may lead to hemolysis. The target predicted by molecular modeling also has limitations, but it provides some references for the study of the potential mechanism of OPD and OPD′ hemolysis.

## 4. Conclusions

In summary, we reported the hemolytic properties of OPD and OPD′ and clarified their differences with regard to their hemolytic behavior *in vivo* and *in vitro*. *In vitro*, we established that hemolysis induced by OPD′ was related to a redox imbalance, and OPD has a certain protective effect. We also put forward some reasonable suggestions for safe clinical use. *In vivo*, we confirmed that both OPD and OPD′ caused hemolysis, and in addition to redox imbalance, proteomics data revealed that lipid metabolic disorder and mitochondrial energy metabolism have extensive participation in hemolysis. Proteomics and molecular modeling provided a comprehensive description of hemolysis-related protein changes and targets, which may benefit further study of the hemolysis mechanisms of OPD and OPD′.

## Figures and Tables

**Table 1:** The putative targets which predicted by SEA.

Index	Affinity threshold (nM)	*p* value	Target name	Target description
1	5	5.25*E*-21	AMYP_HUMAN	Pancreatic alpha-amylase
2	5	1.60*E*-28	AT1A1_CANLF	Sodium/potassium-transporting ATPase subunit alpha-1
3	5	1.00*E*-36	CP125_MYCTU	Steroid C26-monooxygenase
4	5	2.85*E*-09	CP17A_HUMAN	Steroid 17-alpha-hydroxylase/17,20 lyase
5	5	4.51*E*-06	CP17A_RAT	Steroid 17-alpha-hydroxylase/17,20 lyase
6	5	9.68*E*-10	DHCR7_RAT	7-Dehydrocholesterol reductase
7	5	3.91*E*-06	DPOLA_HUMAN	DNA polymerase alpha catalytic subunit
8	5	8.23*E*-06	EBP_HUMAN	3-Beta-hydroxysteroid-delta(8), delta(7)-isomerase
9	5	1.66*E*-07	ERG2_YEAST	C-8 sterol isomerase
10	5	7.00*E*-13	IL2_HUMAN	Interleukin-2
11	5	2.46*E*-08	MRP4_HUMAN	Multidrug resistance-associated protein 4
12	5	6.15*E*-29	NPC1_HUMAN	Niemann-Pick C1 protein

**Table 2:** The top 10 predicted targets based on the shape similarity method.

Rank	Entry	Target name	Organism
1	Q11201	CMP-N-acetylneuraminate-beta-galactosamide-alpha-2,3-sialyltransferase 1	Homo sapiens
2	O54939	Testosterone 17-beta-dehydrogenase 3	Rattus
3	Q9UHC9	Niemann-Pick C1-like protein 1	Homo sapiens
4	Q9NPD5	Solute carrier organic anion transporter family member 1B3	Homo sapiens
5	P60568	Interleukin-2	Homo sapiens
6	P08069	Insulin-like growth factor I receptor	Homo sapiens
7	P31749	Serine/threonine-protein kinase AKT	Homo sapiens
8	P04626	Receptor protein-tyrosine kinase erbB-2	Homo sapiens
9	P30304	Dual specificity phosphatase Cdc25A	Homo sapiens
10	P35372	Mu opioid receptor	Homo sapiens

**Table 3:** The pathways details of the target Q9NPD5.

Index	Function	Term
1	Metabolism (Homo sapiens)	Recycling of bile acids and salts
2	Transport of small molecules (Homo sapiens)	Transport of organic anions

## Data Availability

All data used to support the findings of this study are available from the corresponding author upon request.

## Supplementary Materials

Supplementary Materials See Table S1 in the Supplementary Material for hemolysis-related targets. See Method S1 in the Supplementary Material for detailed proteome analysis conditions.
