# Supplementary material for: Differences in the Hemolytic Behavior of Two Isomers in Ophiopogon japonicus In Vitro and In Vivo and Their Risk Warnings
Source: Oxid Med Cell Longev. 2020 Dec 13;2020:8870656. doi: 10.1155/2020/8870656 (PMC7755485; doi:10.1155/2020/8870656)
Supplement: Supplementary Materials — See Table S1 in the Supplementary Material for hemolysis-related targets. See Method S1 in the Supplementary Material for detailed proteome analysis conditions. [file 8870656.f1.docx]

**Table S1 Hemolysis related targets**

Table S1 Hemolysis related targets.

| Index | Entry | Target name | Organism |
| --- | --- | --- | --- |
| 1 | P00738 | Haptoglobin (Zonulin) | Homo sapiens |
| 2 | P02730 | Band 3 anion transport protein | Homo sapiens |
| 3 | P08237 | ATP-dependent 6-phosphofructokinase, muscle type | Homo sapiens |
| 4 | P09601 | Heme oxygenase 1 | Homo sapiens |
| 5 | P11166 | Solute carrier family 2, facilitated glucose transporter member 1 | Homo sapiens |
| 6 | P13987 | CD59 glycoprotein | Homo sapiens |
| 7 | P15976 | Erythroid transcription factor | Homo sapiens |
| 8 | P30613 | Pyruvate kinase PKLR | Homo sapiens |
| 9 | P48637 | Glutathione synthetase | Homo sapiens |
| 10 | P51811 | Membrane transport protein XK | Homo sapiens |
| 11 | Q13351 | Krueppel-like factor 1 | Homo sapiens |
| 12 | Q9NPD5 | Solute carrier organic anion transporter family member 1B3 | Homo sapiens |
| 13 | Q9Y6L6 | Solute carrier organic anion transporter family member 1B1 | Homo sapiens |
| 14 | P02769 | Serum albumin | Bos taurus |
| 15 | P0C2J9 | Pneumolysin | Streptococcus pneumoniae serotype 4 |
| 16 | P07724 | Serum albumin | Mus musculus |
| 17 | P09598 | Phospholipase C | Bacillus cereus |
| 18 | Q7WZI6, Q7WZI7 | Shiga toxin | Escherichia coli O157:H7 |
| 19 | B2RLK2 | Lys-gingipain | Porphyromonas gingivalis |
| 20 | P49065 | Serum albumin | Oryctolagus cuniculus |
| 21 | P72197 | Lys-gingipain | Porphyromonas gingivalis |
| 22 | P09616 | Alpha-hemolysin | Staphylococcus aureus |
| 23 | P02768 | Serum albumin | Homo sapiens |
| 24 | P02770 | Serum albumin | Rattus |
| 25 | P01501 | Melittin | Apis mellifera |
| 26 | P35747 | Serum albumin | Equus caballus |
| 27 | Q7BQ98 | Shiga toxin subunit B | Shigella dysenteriae |
| 28 | Q8DSW2 | hemolysis inducing protein | Streptococcus mutans UA159 |
| 29 | C5WF02 | conserved hypothetical protein | Streptococcus dysgalactiae subsp. equisimilis GGS_124 |

**Method S1: Detailed proteome analysis conditions**

**Reversed phase chromatography separation (RPLC) conditions**

Liquid chromatography: Agilent 1100 HPLC

Column: Agilent Zorbax Extend-C18 (2.1×150mm, 5μm)

Detection wavelength: UV 210nm and 280nm

Mobile phase A: ACN-H_2_O (2:98, v/v)

Mobile phase B: ACN-H_2_O (90:10, v/v)

Flow rate: 300μL/min

Gradient elution program: 0-8min, 98%A; 8-8.01min, 98%-95%A; 8.01-48min, 95%-75%A; 48-60min, 75%-60%A; 60-60.01min, 60%-10%A; 60.01-70min, 10%A; 70-70.01min, 10%-98%A; 70.01-75min, 98%A.

Dried samples were harvested from 8min to 60min and elution buffer were collected in every minute and numbered from 1-15 with pipeline. The separated peptides were lyophilized for MS detection.

**Proteome analysis conditions (MS Detection)**

The sample was separated by a pre-column Acclaim PepMap100 (100μm×2cm) and a Acclaim PepMap RSLC (75μm×15cm) column in turn.

Mobile phase A: FA-H_2_O (99.9:0.1, v/v)

Mobile phase B: ACN-H_2_O-FA (80:19.9:0.1, v/v/v)

Gradient elution program: 0-40min, 5%-30%B; 40-54min, 30%-50%B; 54-55min, 50-100%B; 55-60min, 100%B.

Full MS scans were acquired in the mass range of 300–1600 m/z with a mass resolution of 70000 and the AGC target value was set at 1e6. The ten most intense peaks in MS were fragmented with higher-energy collisional dissociation (HCD) with NCE of 32. MS/MS spectra were obtained with a resolution of 17500 with an AGC target of 2e5 and a max injection time of 80ms. The Q-E dynamic exclusion was set for 30.0 s and run under positive mode.
